# Inverse dynamics of mechanical multibody systems: An improved algorithm that ensures consistency between kinematics and external forces

**DOI:** 10.1371/journal.pone.0204575

**Published:** 2018-09-28

**Authors:** Herre Faber, Arthur J. van Soest, Dinant A. Kistemaker

**Affiliations:** 1 Department of Human Movement Sciences, Faculty of Behavioural and Movement Sciences, Vrije Universiteit Amsterdam, Amsterdam Movement Sciences, Amsterdam, The Netherlands; 2 Faculty of Health, Nutrition and Sports, The Hague University of Applied Sciences, The Hague, The Netherlands; University of Zaragoza, SPAIN

## Abstract

Inverse dynamics is a technique in which measured kinematics and, possibly, external forces are used to calculate net joint torques in a rigid body linked segment model. However, kinematics and forces are usually not consistent due to incorrect modelling assumptions and measurement errors. This is commonly resolved by introducing ‘residual forces and torques’ which compensate for this problem, but do not exist in reality. In this study a constrained optimization algorithm is proposed that finds the kinematics that are mechanically consistent with measured external forces and mimic the measured kinematics as closely as possible. The algorithm was tested on datasets containing planar kinematics and ground reaction forces obtained during human walking at three velocities (0.8 m/s, 1.25 and 1.8 m/s). Before optimization, the residual force and torque were calculated for a typical example. Both showed substantial values, indicating the necessity of developing a mechanically consistent algorithm. The proposed optimization algorithm converged to a solution in which the residual forces and torques were zero, without changing the ground reaction forces and with only minor changes to the measured kinematics. When using a rigid body approach, our algorithm ensures a consistent description of forces and kinematics, thereby improving the validity of calculated net joint torque and power values.

## Introduction

In the field of biomechanics, inverse dynamics analysis is commonly used to investigate aspects of the mechanics, energetics and control of movement. An inverse dynamics analysis is typically based on measurement of the kinematics of the body segments, often complemented with measurement of selected external forces (e.g. the ground reaction force). Using these data, the net joint torques and net joint reaction forces are calculated using Newton’s equations of motion applied to a model containing a (chain of) rigid segments [1]. Classically, these equations are solved consecutively for each body segment, starting at a segment for which the number of unknowns matches the number of equations. Although inverse dynamics is straightforward, it is not without problems [2–4]. First, classical inverse dynamics assumes idealized pin joints and rigidity of body segments, which in reality don’t occur. Second, measurement errors in kinematic data caused by noise and skin artifacts lead to incorrect joint centre locations, velocities and accelerations and thereby to errors in net joint torques. Third, the anthropometric parameters for a particular subject (such as segment masses, mass center locations and segmental inertia) are typically estimated on the basis of a limited number of anthropometric characteristics in combination with results of cadaver studies [5]. Their values will deviate from the actual values, resulting in errors in net joint torques. In an attempt to mitigate these problems, external forces are often accurately measured and used as an additional input in the inverse dynamics analysis, thereby improving on its quality. However, using both measured kinematics and measured external forces in an inverse dynamics analysis introduces a new problem, since they will typically be inconsistent due to the aforementioned problems. This new problem is commonly formulated as follows [6]: the net joint torques obtained from an inverse dynamics analysis starting at the unconstrained end of a chain of segments (e.g. the hands of a free standing person) and ending at the feet are different from those obtained when the analysis is started at the feet. In more formal terms the new problem is that the system of equations of motion for a complete linked segment model is overdetermined. One way to evade the inconsistency is to ignore information, i.e. to discard the equations of motion, about the mechanics of the last segment. Another way is to use all equations, which results in a residual force and torque typically applied at the last segment by an unspecified actor in the environment. In fact, both will result in the same values for the joint torques. The residual force and torque compensate for the measurement errors in kinematics and incorrect model assumptions, but do not exist in reality. Their values can actually be considered as an indication of the validity of the calculated joint forces and torques. Furthermore, the residual force and torque do perform mechanical work that does not exist in reality and therefore may compromise energetic analyses.

In sum, in an inverse dynamics analysis, assuming a rigid body linked segment model as a basis, kinematics are in general inconsistent with measured external forces, i.e. result in nonzero residual forces and torques. The question then arises how the inconsistency can be reduced or, even better, completely removed under the assumption of segment rigidity. This can be accomplished in three ways: by adjusting i) the (time-invariant) anthropometric data, ii) the kinematics or iii) the external force(s). Several studies have used (combinations of) these ways in an attempt to reduce or remove the residual forces and torques. For example, Vaughan [7] optimized body segment parameters to minimize residual forces and torques. A complete removal of the residual forces and torques will in general not be possible since the number of anthropometric variables is typically smaller than the number of time nodes in the analysis. Delp et al [8] optimized model mass parameters and kinematics to reduce residual forces and torques, but did not succeed in completely removing the residual forces and torques. De Groote et al [9] adjusted the kinematic data by employing a Kalman smoother that used the complete kinematic dataset. Even though this method improved the estimate of joint kinematics, it did not address the problem of the residual forces and torques. Chao and Rim [10], using an optimal control approach, optimized joint torques to minimize the squared differences between measured and calculated segment angles. However, ground reaction forces were not investigated and hence this method did not remove the residual forces and torques. Thelen and Anderson [11] calculated translational accelerations of the pelvis and low back angles assuming that the ground reaction forces and all other generalized coordinates were well represented by measurements. Integration of the accelerations over time yielded the pelvis and low back kinematics with residuals removed. Boundary values for pelvis and low back were subsequently optimized to minimize the difference between measured and calculated kinematics. However, it is highly unlikely to find the optimal kinematic profile by optimizing the kinematics of only two instead of all segments. Van Soest [12], Kuo [3] and van den Bogert and Su [13] optimized joint torques using all segments for each time node separately such to find a least squares solution to the overdetermined set of equations of motion, but this does not remove the residual forces and torques. Cahouet et al [14] composed a set of equations of motion for each time node and a centered finite difference scheme relating angular acceleration and position. The resulting overdetermined set of equations was solved using a least squares method. Their solution resulted, in the presence of measurements errors, in an inconsistency between position, force measurements and angular accelerations. Remy and Thelen [15] adjusted measured ground reaction forces, ground reaction torques and segment angular accelerations during walking, which yielded a consistent description of these quantities for each separate time node. However, as stated, this algorithm required adjustment of the ground reaction force and torque, which is in fact similar to applying residual forces and torques at the feet instead of the trunk. These studies [3,12–15] combined, show that any attempt to improve on inverse dynamics by optimizing for separate time nodes either leads to an inconsistent mechanical description or to an undesired shift of the residual force and torque to a different segment. Mazzà and Cappozzo [16] were one of the first to solve this problem by performing an optimization over the whole time-series, while successfully removing the residual forces and torques. They optimized segment angles, which were used in a top-down approach to minimize the root mean square error between measured and calculated ground reaction force. Among the input for their algorithm were segment angles at the start and end of the movement which were constrained to be reproduced by their algorithm. However, they made no attempt to ensure that the intermediate calculated and measured kinematics were similar. This was improved upon by Riemer and Hsiao-Wecksler [17] who also optimized segment angles to minimize the ground reaction force root mean square error. They introduced inequality constraints for the intermediate segment angles based on data from the literature to create a range in which the optimized segment angles could be found. Riemer and Hsiao-Wecksler [18] expanded the method of Riemer and Hsiao-Wecksler [17] by adding body segment parameters to the variables to be optimized. It was shown, using an idealized dataset, that reconstruction of net joint torques could benefit significantly from optimizing body segment parameter values. However, one problem remains in their approach. Assume *n* degrees of freedom for a chain of *n* segments connected by pin joints representing the body, *N* time nodes and also assume that the external forces are chosen such to perfectly fit the measured external forces. In the planar case, this yields 2(*n*-1) joint force components, *n*-1 joint torques and *n* segment angles summing up to 4*n*-3 variables to be optimized for each time node. Since there are three equations of motion for each time node, yielding 3*n* equations, the complete system has 3*n*N equations and (4*n-3)*N unknowns. If the number of degrees of freedom is three, such a system is determined. Overdeterminacy occurs for values of *n* between zero and three, whereas underdeterminacy always occurs for values of *n* larger than three. This means that in applications with more than three degrees of freedom, like the lifting example given by Riemer and Hsiao-Wecksler [17], there are many optimal kinematic profiles, i.e. kinematic profiles yielding a perfect fit of the calculated and measured ground reaction force. The method by Riemer and Hsiao-Wecksler is not guaranteed to find the optimal kinematic profile that best fits the measured kinematics. The under determinacy could therefore be used to find the unique solution that leads to an optimal fit between measured and optimized kinematics, while removing the residuals completely.

To conclude, no inverse dynamics method is currently available in which i) all residual forces and torques are removed, ii) segment angles at all time nodes are optimized together, and iii) the problem is defined such that it always produces a unique solution, i.e. it results in minimal adaptation of the kinematics while the external forces are not accommodated. The purpose of this study was to develop an algorithm that improves on inverse dynamics while meeting these demands. To show the significance of the inconsistency between kinematics and external forces, the magnitudes of the residual force and torque values of a classical inverse dynamics analysis were obtained from a dataset concerning human gait. The resulting optimization algorithm was evaluated by applying it to the same dataset, comparing the results (kinematics and joint torques) to those obtained using a classical inverse dynamics analysis. In the example application, the dataset consisted of the sagittal plane coordinates of markers attached to body segments, sagittal plane ground reaction force data (including point of application) and segment parameter values. After optimization of the dataset, the measured ground reaction force and kinematics were fully consistent.

## Results

### Residual force and torque for the classical inverse dynamical analysis

**Fig 1:**
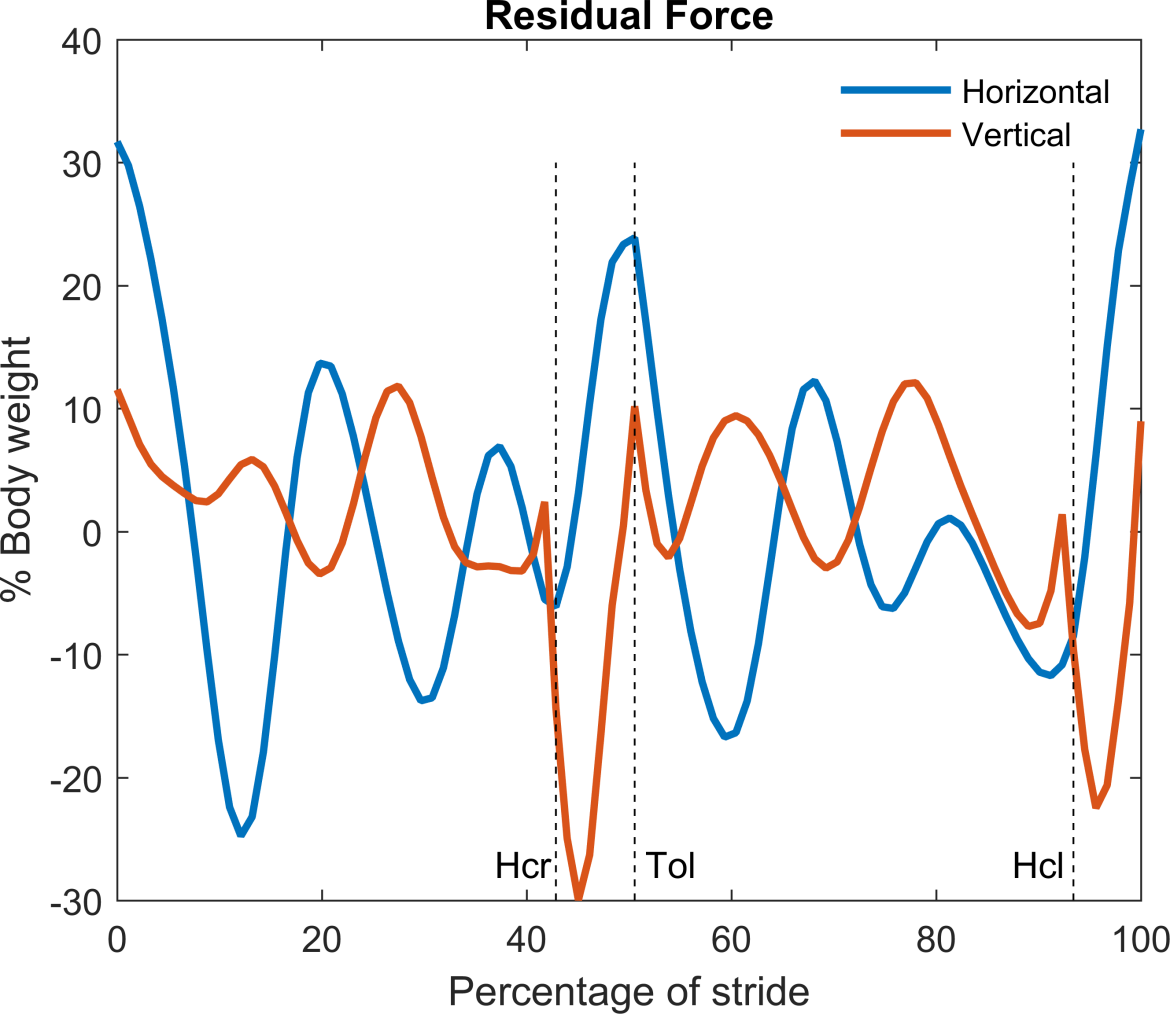
Residual force resulting from a classical inverse dynamics analysis on the typical example. Zero percent of the stride coincides with right toe off for all time series. Force was expressed in percentage of body weight. The analysis shows considerable residual forces to enforce consistency between kinematics and measured ground reaction forces. Zero crossings indicate the time nodes where measured ground reaction forces and kinematics were ‘accidentally’ consistent. Hcr: heel contact right foot, Tol: toe off left foot, Hcl: heel contact left foot. Right toe off is defined as the onset of the stride.

We performed a classical inverse dynamics analysis on one complete stride of a subject walking at 1.8 m/s, which yielded the residual forces on the trunk (see Fig 1). This trial will be referred to as the typical example. The onset of the stride was defined by toe off of the right leg. Positive *x*- and *y*-forces were defined as in the walking direction (forward) and upward, respectively. From Fig 1 it can be observed that in particular the horizontal component of the residual force at the trunk was substantial. Note again that these forces do not exist in reality.

**Fig 2:**
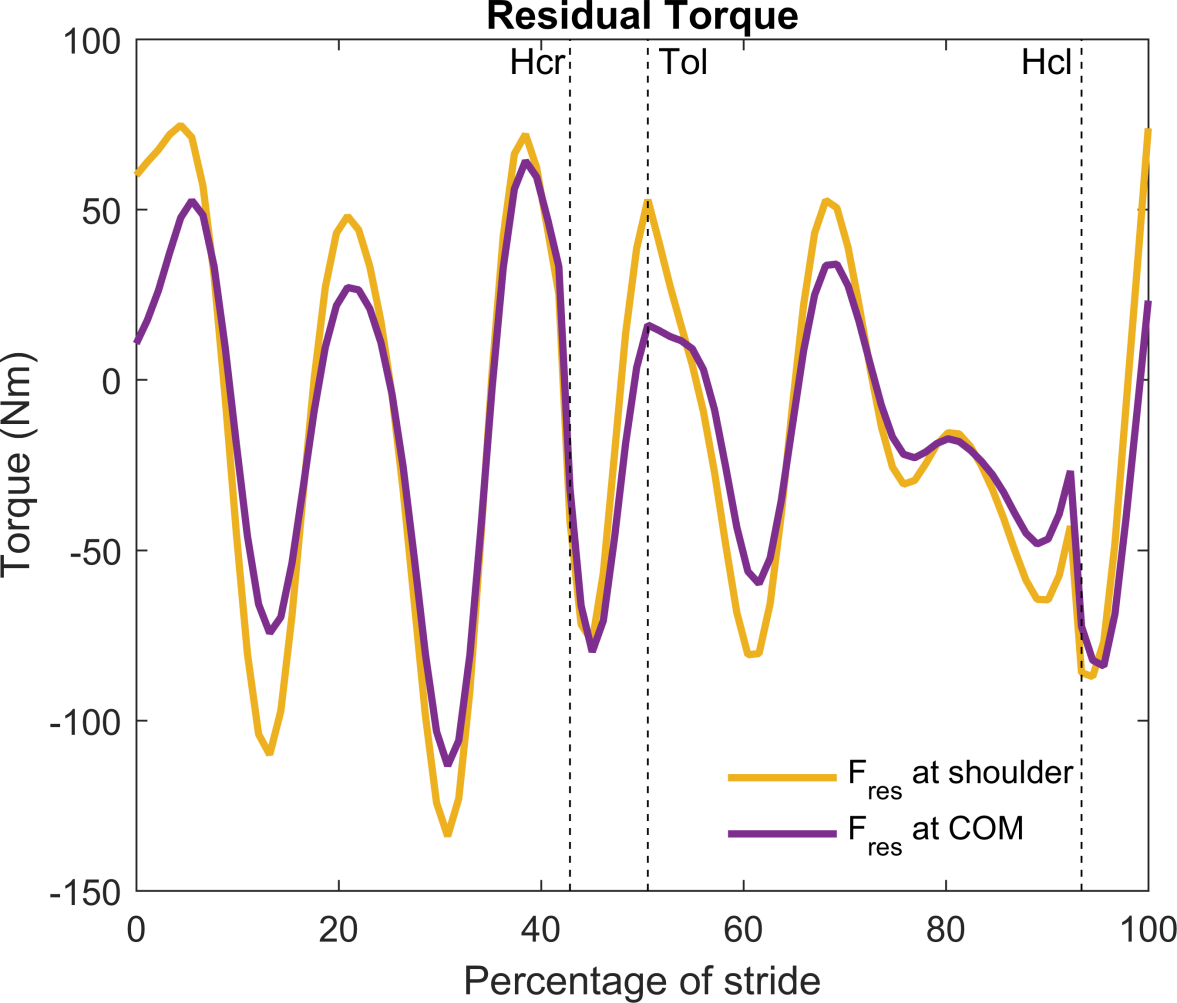
Residual torque on the trunk of the typical example. To show the effect on the residual torque, the residual force (*F*_*res*_) was assumed to either apply at the shoulder or at the center of mass of the trunk. Clockwise torques were defined positive. Hcr: heel contact right foot, Tol: toe off left foot, Hcl: heel contact left foot.

The values of the residual force were directly related to the inconsistency between measured ground reaction forces and acceleration of the body’s center of mass and hence were not affected by its presumed point of application. In contrast, the value of the residual torque was affected by the (arbitrarily chosen) point of application of the residual force. To illustrate this, two classical inverse dynamics analyses were performed. In the first, the residual force was applied at the shoulder, while in the second it was applied at the trunk’s center of mass (Fig 2). Marked differences for the residual torque value were observed between these two analyses. This indicates that the value of the residual torque by itself is meaningless. An interaction between the residual force and torque was observed. The relatively large positive horizontal residual force at the shoulder, for example at t = 0 in Fig 1, yielded a negative (flexion) torque at the trunk. This was compensated for by an opposite (positive) residual torque applied at the trunk (Fig 2), which largely explained the in phase behavior of the horizontal residual force component and the residual torque.

### Optimization results

**Fig 3:**
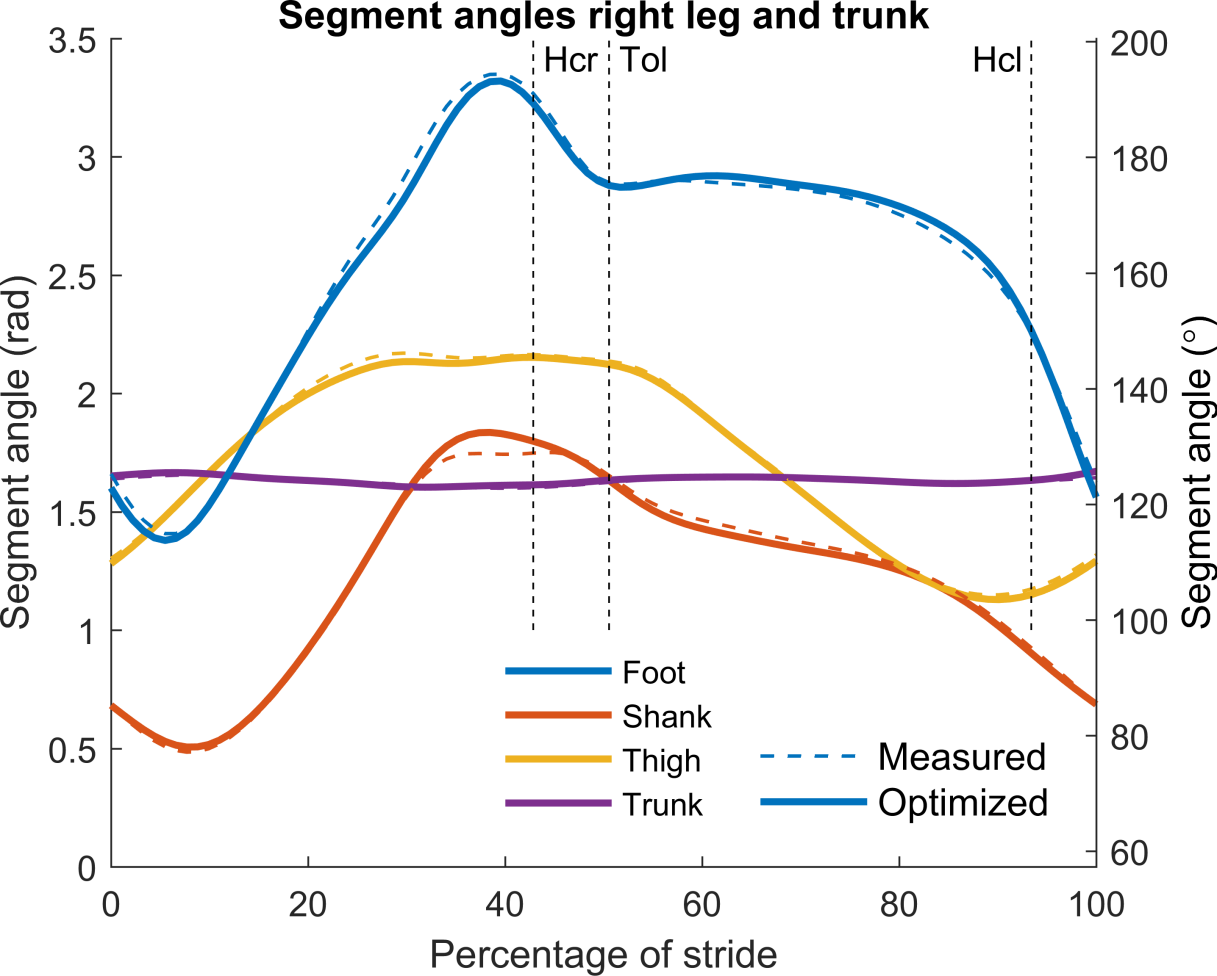
Typical example of segment angles during a stride. The respective segments are denoted by color. Segment angles before and after optimization are denoted by dashed and solid lines respectively. For definitions of the segment angles, see the Materials and Methods section. Hcr: heel contact right foot, Tol: toe off left foot, Hcl: heel contact left foot.

**Fig 4:**
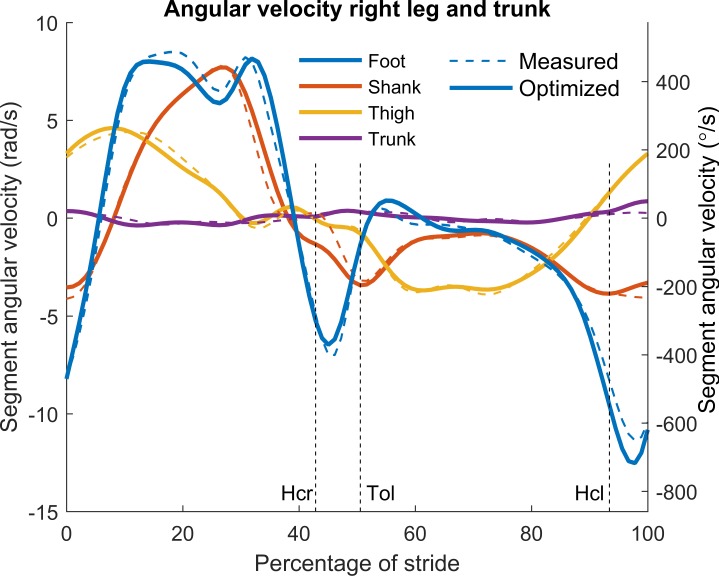
Typical example of segment angular velocities during a stride. The respective segments are denoted by color. Angular velocities before and after optimization are denoted by dashed and solid lines respectively. Hcr: heel contact right foot, Tol: toe off left foot, Hcl: heel contact left foot.

A rigid body linked segment model was defined to describe the kinematics, forces and torques during a set of walking trials. The kinematic profiles were found by minimization of the sum of all the Euclidean distances between measured and model skin marker positions. Removal of the residual forces and torques was ensured by adding the equations of motion of all segments with no residuals as equality constraints. The resulting single core optimization of one stride took on average 2 minutes on an Intel i7-4770 (3.4 GHz) processor, using the measured data as the initial guess. The solutions always converged and the residual force and torque were completely removed. In Figs 3 and 4 the measured and optimized segment angles and angular velocities of the right leg and trunk were compared for the same typical example used for Figs 1 and 2. The results showed that only small changes in optimized angles were necessary to completely remove the residual forces and torques. We then performed comparisons for 61 strides in nine different subjects walking at three different speeds (0.8, 1.25 and 1.8 m/s) and calculated root mean square (RMS) values for the differences between trunk and lower segment angles before and after optimization. Table 1 provides the average RMS values of the foot, shank, thigh and trunk angles. These values indicate that on the whole, like with the typical example, only small changes in kinematics were required to completely remove the residual forces and torques.

**Table 1:** Average RMS values of the segment angles with standard deviations.

Segment angle	*Average RMS*_*q*_ (sd)	*Maximum deviation*
**Right foot**	0.056 (0.019)	0.276
**Right lower leg**	0.024 (0.011)	0.153
**Right upper leg**	0.025 (0.012)	0.158
**Trunk**	0.022 (0.011)	0.222

These values provide an indication of the difference in segment angles in radians before and after optimization. Subscript *q* refers to the degrees of freedom (segment angles) as explained in the Materials and Methods section.

**Fig 5:**
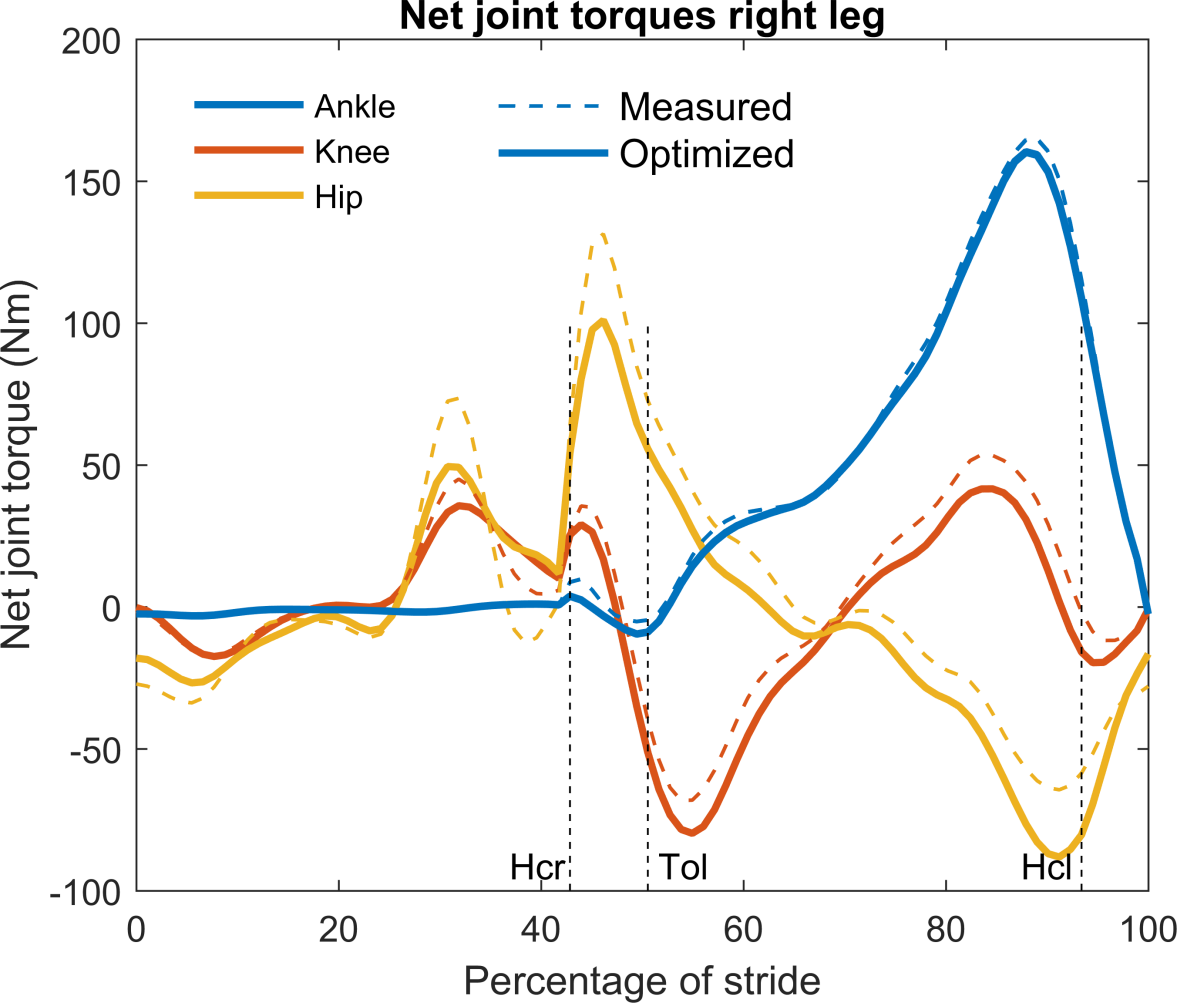
Net joint torques of the typical example before and after optimization. Optimized and classical net joint torques were similar. Thin dashed lines indicate joint torque values prior to optimization. Thick solid lines indicate joint torque values after optimization. Positive values denote plantar flexion, knee flexion and hip extension torques. Hcr: heel contact right foot, Tol: toe off left foot, Hcl: heel contact left foot.

We also compared the distances between the markers, located at the joints (indicated in Table 2), before and after optimization. RMS values for the markers (*RMS*_*s*_ in Table 2) were in the order of 1 cm which also indicated good agreement between measured and optimized kinematics. Net joint torques were calculated before and after optimization (Fig 5) and the differences were quantified by a relative measure as shown in Table 2. RMS values of the relative differences for the joint torques before and after optimization indicated larger differences than for the measured and optimized joint angles. Both sets of torques showed similar patterns, although hip torque peak values before and after optimization were substantially different.

**Table 2:** RMS values of the marker positions and net joint torques.

Marker	Average *RMS*_*s*_ (sd)	*Maximum distance markers*	*Average RMS*_*T*,*rel*_ (sd)	*Maximum range RMS*_*T*,*rel*_
**Right fifth MTP joint**	0.7 (0.4)	4.8	*NA*	*NA*
**Right ankle**	1.0 (0.4)	4.7	0.127 (0.157)	0.8
**Right knee**	1.1 (0.4)	5.8	0.419 (0.198)	2.2
**Right hip**	1.3 (0.7)	10.1	0.480 (0.255)	2.6
**Right shoulder**	1.2 (0.7)	*15*.*0*	*NA*	*NA*

First column: RMS values of the difference in marker positions (*RMS*_*s*_) before and after optimization (cm). Second column: maximum distance between markers before and after optimization (cm). Third column: relative differences in net joint torques before and after optimization (*RMS*_*T*,*rel*_). Calculation of these values was performed according to Eq (5) of the Methods section. Fourth column: maximum relative deviations of *RMS*_*T*,*rel*_. *NA*: not applicable. MTP joint: metatarsophalangeal joint.

**Fig 6:**
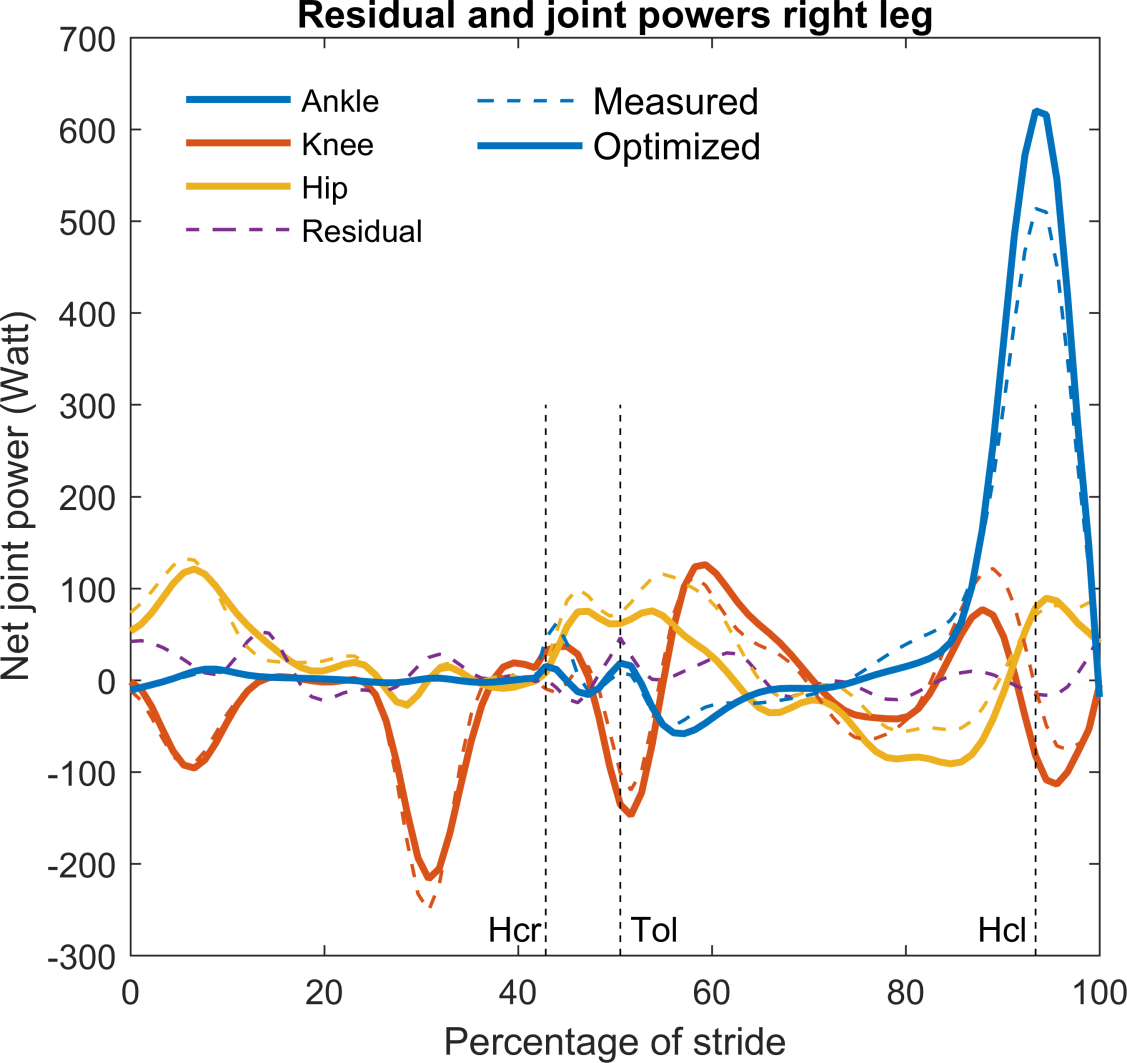
Net joint and residual power values of the typical example before and after optimization. Residual power is the sum of the power of the residual torque and force at the shoulder before optimization. Hcr: heel contact right foot, Tol: toe off left foot, Hcl: heel contact left foot.

The net joint powers depicted in Fig 6 both before and after optimization were shown to be substantially larger than the residual power (when the residual force was applied at the shoulder) for the typical example. The absolute peak power of the residual force was in the order of 50 Watt. Removing the residual force and torque led to a maximum adjustment of the ankle power (at 90 percent of the stride) in the order of 100 Watt.

## Discussion

In a classical inverse dynamics analysis, based on a rigid linked segment model, measured kinematics and external forces are in general not mechanically consistent. In this study, an algorithm was developed to remedy this by modifying the measured kinematics as little as possible such that the resulting optimized kinematics are mechanically consistent with measured external forces. As an example, this algorithm was applied to a dataset of human walking containing 2D joint positions. Our analyses show that the algorithm was capable of completely removing the residual forces and torques during a stride with minor changes to the measured kinematics, while leaving the measured ground reaction forces unchanged. As a result, joint torque profiles before and after optimization showed similar patterns.

The example used in this study was a 2D representation of walking. However, we stress that it is straightforward to extend the algorithm in several directions. First, we note that extension to 3D is straightforward. For example, in walking experiments with ground reaction force and 3D measurement of kinematics, three residual force components and three residual torque components will arise at the trunk. These can be treated the same way as in the planar case. However, due to increased model complexity in 3D applications, it should be established in future work how this affects the calculation time of the optimization. Second, as mentioned in the introduction, several methods exist in which body segment parameter values are added to the variables to be optimized. These were not included in our algorithm because we focused on altering the kinematics and its effect on the residual force and torque values. However, including body segment parameter values and imposing reasonable bounds is a relatively simple extension, which can contribute to improving inverse dynamics analysis. Third, human walking is an example of a (nearly) periodic movement. Conceivably, researchers may want to impose strict periodicity on such a movement. In that case, the external forces should be (minimally) adjusted such that the cycle average of the sums of all forces and torques equal zero. Also, constraints should be added to enforce equal positions and velocities at the start and end of the cycle.

Summarizing, a straightforward algorithm was developed that completely removed residual forces and torques in an inverse dynamics analysis. It was found that small adjustments to the kinematics only, in the order of 1 cm marker displacements, were sufficient to obtain a consistent mechanical description. The algorithm provides a clear improvement over current methods in calculating net joint torques and it should, in our opinion, therefore be included in any rigid body inverse dynamics analysis.

## Materials and methods

### Description of the proposed algorithm

For any application, depending on the analyzed movement, the first step is to define a model consisting of *m* rigid bodies that are connected by joints, represented by kinematic constraints imposed on the kinematics of the rigid bodies. Next, values are assigned to the time-invariant properties of each of these segments (length, center of mass position in a local frame of reference and inertial properties). When the model has *n* degrees of freedom, *n* generalized coordinates *q* suffice to fully describe the position of the model at a particular time node *i*. Thus $Q=\bar{q}\left(t\left(i\right)\right)=\left(q_{1}\left(t\left(i\right)\right),q_{2}\left(t\left(i\right)\right),\ldots ,q_{n}\left(t\left(i\right)\right)\right)$ contains the full description of the position of the system at time *t(i)*. If the total number of time nodes considered is *N*, then the position of the system over time is completely described by an *N*x*n* matrix *Q*, containing the $\bar{q}\left(t\left(i\right)\right)$ as rows. This implies that all other kinematic variables of interest can be calculated from *Q*. In particular, we calculate the matrix *Z*, containing the Cartesian coordinates of the centers of mass of all segments at all times, the matrix *P* containing the Cartesian coordinates of all joint centers and the matrix *S* containing the predicted positions of the skin markers used in the kinematics registration. Note that the latter contain time-invariant coordinate values relative to a segment-fixed frame of reference, which can be obtained by calibration measurements. Given these matrices, the relevant second derivatives with respect to time are approximated using central differences:

$$\ddot{\bar{q}}\left(t\left(i\right)\right)=\frac{\bar{q}\left(t\left(i‑1\right)\right)‑2\cdot \bar{q}\left(t\left(i\right)\right)+\bar{q}\left(t\left(i+1\right)\right)}{{\left(t\left(i+1\right)‑t\left(i\right)\right)}^{2}}$$ (1)

**Fig 7:**
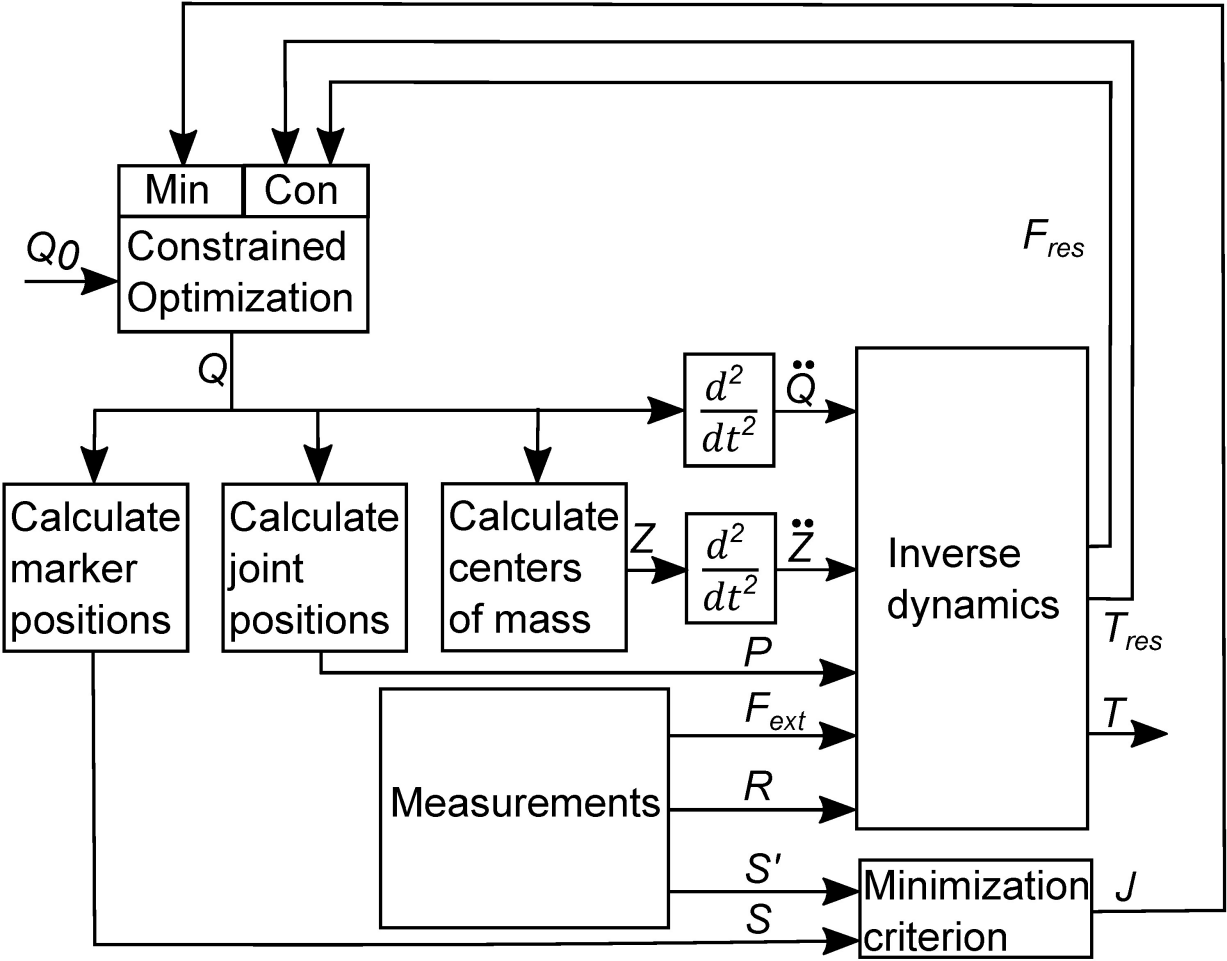
Scheme of the proposed algorithm. The optimization starts by providing an initial guess for the matrix *Q*_*0*_ that contains the values for the degrees of freedom at each time node, calculated from the measured marker coordinates. The optimizer generates a modified version of *Q*. Using rigid body kinematics and numerical differentiation, the kinematic variables relevant for inverse dynamics are calculated. In the inverse dynamics block, net joint torques and forces (including residual forces and torques) are calculated on the basis of these kinematic variables, in combination with the measured external forces *F*_*ext*_, and their points of application *R*. The residual forces *F*_*res*_ and torques *T*_*res*_ and the predicted marker positions are fed back to the optimizer, which updates *Q* such that, ultimately, the residuals are zero and the sum of the weighed squared Euclidian distances (*J*) between predicted (*S*) and measured (*S*’) marker positions is minimal.

In general terms, the optimization problem is to minimize the sum of the weighed squared Euclidian distances between the segment model markers *S* and the corresponding experimentally observed markers *S*’, without the introduction of residual forces and torques. A matrix *R*, containing the measured points of application of the external forces is fed into an inverse dynamics analysis, which yields the residual forces (*F*_*res*_) and the residual (*T*_*res*_) and net joint torques (*T*). The constrained optimization problem is solved by the proposed algorithm as indicated in Fig 7.

Formally, the optimization problem can be summarized as follows:

Find *Q* that minimizes:

$$J={\sum_{i=1}^{N}\sum_{k=1}^{c}w_{j}|{\bar{s}}_{j}\left(t\left(i\right)\right)-\bar{s}{'}_{j}\left(t\left(i\right)\right)|}^{2}$$ (2)

Subject to:

$$T_{res,m}\left(t\left(i\right)\right)=0\;\mathrm{and}\;{\bar{F}}_{res,m}\left(t\left(i\right)\right)=0,\;\mathrm{for}\;\mathrm{all}\;i.$$

Where:

$${\bar{s}}_{j}\left(t\left(i\right)\right):\;\mathrm{vector}\;\mathrm{containing}\;\mathrm{coordinates}\;\mathrm{of}\;\mathrm{the}j‑\mathrm{th}\;\mathrm{calculated}\;\mathrm{marker}\;\mathrm{at}\;\mathrm{time}\;\mathrm{node}i$$

$$\bar{s}{'}_{j}\left(t\left(i\right)\right):\;\mathrm{vector}\;\mathrm{containing}\;\mathrm{coordinates}\;\mathrm{of}\;\mathrm{the}j‑\mathrm{th}\;\mathrm{measured}\;\mathrm{marker}\;\mathrm{at}\;\mathrm{time}\;\mathrm{node}i$$

*w*_*j*_: an optional weight for the relative contribution of the marker to the minimization criterion

*T*_*res*,*m*_*(t(i))*: *m*-th residual torque at time node *i*

$${\bar{F}}_{res,m}\left(t\left(i\right)\right):\;m‑\mathrm{th}\;\mathrm{residual}\;\mathrm{force}\;\mathrm{at}\;\mathrm{time}\;\mathrm{node}i$$

*N*: number of time nodes

*c*: number of markers

As stated before, the segments’ centers of mass accelerations are calculated by direct numerical differentiation of the center of mass position, which are functions of the generalized coordinates. A different method that should produce similar results, would be to express the accelerations in terms of the generalized coordinates and their infinitesimal derivatives, subsequently numerically differentiate the generalized coordinates and replace the infinitesimal derivatives by the numerical analogues. This was found to result in a slightly lower value for the minimization criterion *J*, but introduced numerical instabilities in the form of high frequency oscillations of the calculated generalized coordinates. This was never observed with the direct differentiation as indicated in Fig 7.

### Application to human walking

To test the optimization algorithm, we applied it to human walking. To do so, we measured the kinematics and ground reaction forces during shod walking of nine subjects (all female). This study was approved by the local ethics committee (Ethische Commissie Bewegingswetenschappen) and all procedures were carried out with adequate understanding and after written consent of the subjects. Age was 23.6 ± 1.4 yr (average ± SD). Height was 1.75 ± 0.05 m and body mass 66.1 ± 4.9 kg. Subjects walked for five minutes on an instrumented split belt treadmill (R-Mill, Forcelink, Culemborg, The Netherlands) to get accustomed to the experimental situation. Subjects were instructed to walk with their left and right foot on the separate belts of the treadmill. Subsequently they walked at three different speeds (0.5 m/s, 1,25 m/s and 1.8 m/s) for five minutes at each speed. Optotrak CERTUS Position Sensors (Northern Digital, Waterloo, Ontario) were used to collect kinematics at a sample rate of 100 Hz. In this study single markers were placed at both sides of the body at the fifth metatarsophalangeal joint, the lateral malleolus, the lateral knee epicondyle, greater trochanter and acromion; it was assumed that the marker positions represented the positions of the corresponding joint axes. Raw data was filtered using a zero lag 4^th^ order low-pass filter with a cut-off frequency of 10 Hz. Only sagittal plane projections were used in this study. Ground reaction forces (*F*_*GR*_) were measured at a sample frequency of 200 Hz using two force plates embedded in the treadmill. Raw *F*_*GR*_ data was filtered using a zero-lag 4^th^ order low-pass filter with a cut-off frequency of 20 Hz from which the center of pressure (*r*) was calculated for each foot. Subsequently *F*_*GR*_ and *r* data were down sampled to 100 Hz to match the Optotrak sample rate.

61 strides were selected from the data. All nine subjects and all three velocities were represented in this selection. Start and end of one complete stride was defined by toe off of the right foot (first sample with *F*_*GR*_ equal to zero). Only strides with two distinct swing phases, where *F*_*GR*_ was very close to zero, were selected by visual inspection. Kinematics and *F*_*GR*_ were used both for a classical inverse dynamics and as input for the optimization algorithm. The two hips and two shoulders were lumped together and regarded as one joint. Anthropometric parameters were obtained from Winter [5]. To assess the effect of the point of application of the residual force (before optimization) on the residual torque value, it was applied on the trunk in a classical inverse dynamics analysis at two different positions: the shoulder and the trunk’s center of mass. For each case the residual torque was calculated.

**Fig 8:**
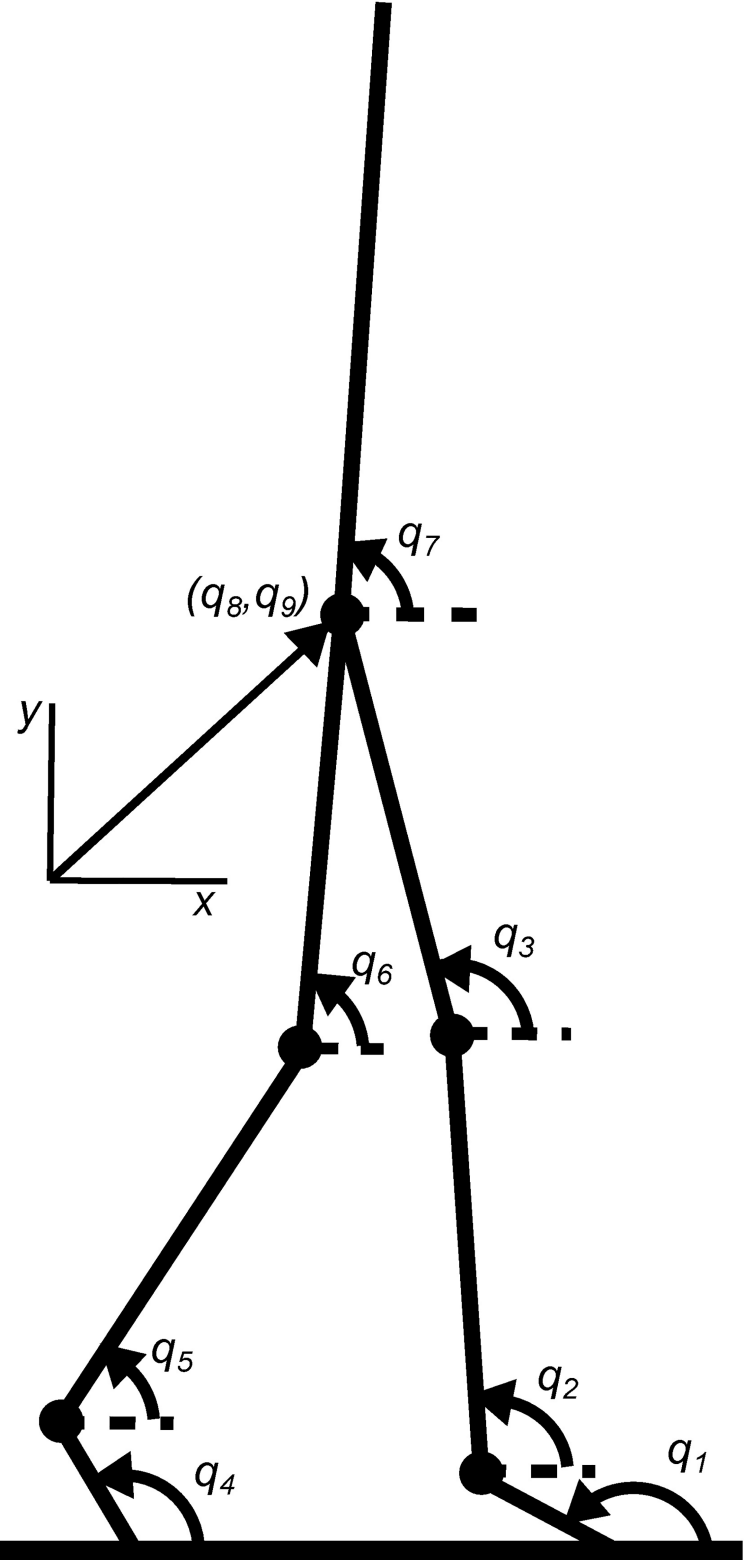
The mechanical model used in the evaluation of the proposed algorithm, considering sagittal plane walking. The model consists of seven rigid segments connected with pin joints. It has nine degrees of freedom. Angular coordinates used to describe the degrees of freedom are indicated by *q*_*1*_- *q*_*7*_. The remaining two degrees of freedom are described by the position of the hip (*q*_*8*_,*q*_*9*_).

For the optimization, the subjects in this study were modeled as a system of 7 rigid segments moving in a vertical sagittal plane, with pin joints connecting the segments and no kinematic constraints between the feet and the walking surface (see Fig 8). This model has nine degrees of freedom, which were described by generalized coordinates *q*_1_..*q*_9_ as defined in Fig 8. Seven coordinates are segment angles; the x- and y-coordinates of the hip were chosen to specify the model’s position in the global frame of reference. In this case, no calibration measurements were required to define the time-invariant positions of the markers relative to a local frame of reference, as the markers were assumed to be placed at the joint axes. Relative contributions *w*_*j*_ of the differences in optimized and measured marker positions to the minimization criterion *J* were all set to 1. Segment lengths were calculated as the average value of the relevant inter-marker distances.

**Fig 9:**
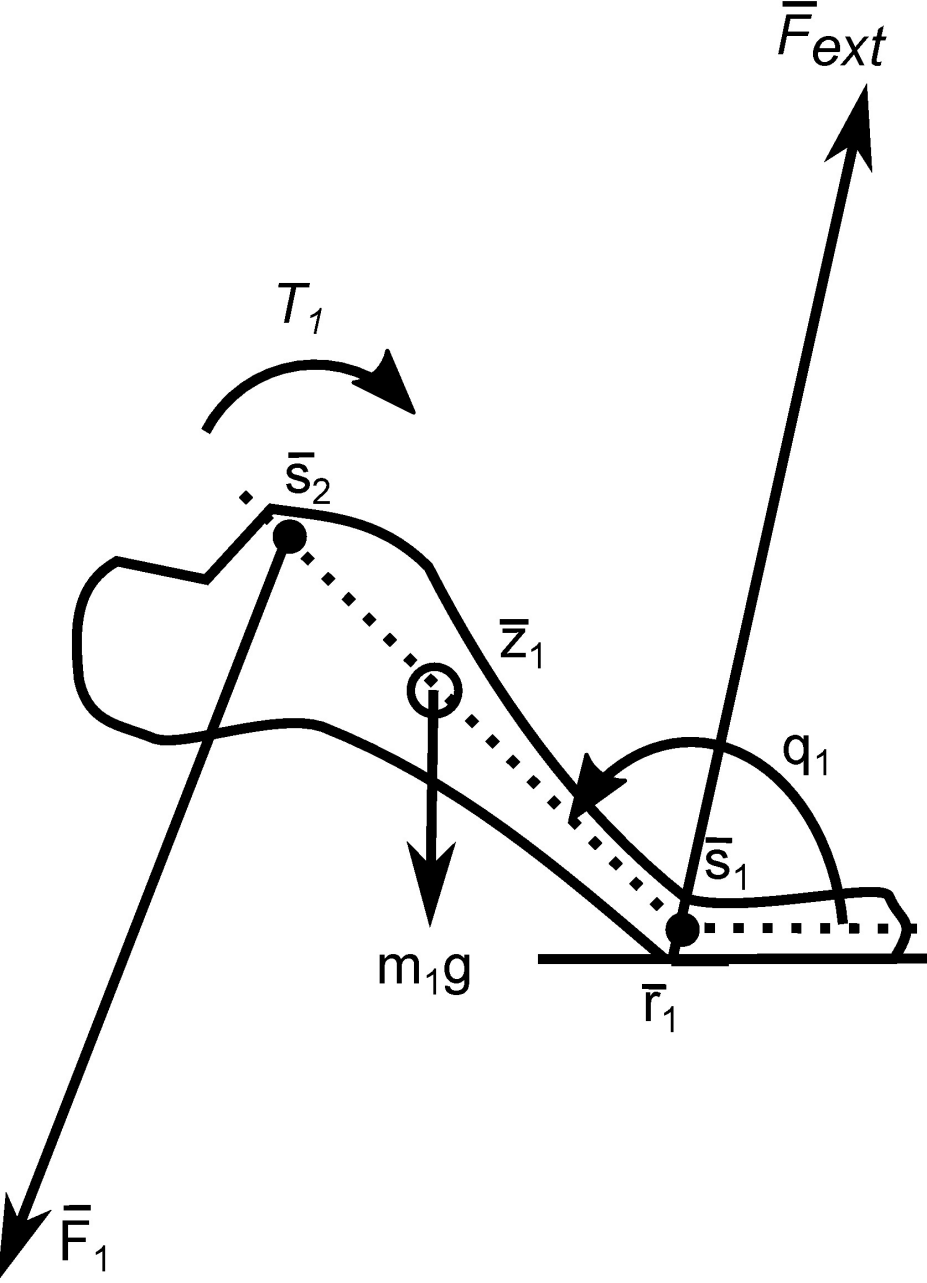
Mechanical model of the foot as applied in inverse dynamics. The external force ${\bar{F}}_{ext}$, its point of application ${\bar{r}}_{1}$ and the markers ${\bar{s}}_{1}$ and ${\bar{s}}_{2}$ are input of the analysis. Torques of ${\bar{F}}_{ext}$, the net ankle force ${\bar{F}}_{1}$ around the foot’s center of mass, the net ankle torque *T*_*1*_ and the force of gravity *m*_*1*_*g* are inserted into Newton’s equations of motion and solved for ${\bar{F}}_{1}$ and *T*_*1*_. These are subsequently reversed according to Newton’s third law and input for the same procedure on the shank.

Fig 9 shows the first segment in the inverse dynamics analysis. The external force ${\bar{F}}_{ext}$ and its point of application ${\bar{r}}_{1}$ have been measured. The kinematics have been measured before or updated by the optimizer during optimization. Application of Newton’s equations of motion yields three equations, which are solved for the net ankle torque *T*_*1*_ and the net ankle force ${\bar{F}}_{1}$. These are subsequently reversed according to Newton’s third law and at the ankle joint applied to the shank which yields the net knee torque and force etc.

The constrained optimization problem was solved using the function *fmincon* embedded in Matlab R2013a. To evaluate the proposed algorithm, the kinematics were compared in terms of the root mean square value of the difference between the generalized coordinates before and after optimization, respectively *q’*_*j*_*(t(i))* and *q*_*j*_*(t(i))*:

$$RMS_{q,j}=\sqrt{\frac{1}{N}\cdot \sum_{i=1}^{N}{\left(q_{j}\left(t\left(i\right)\right)-q{'}_{j}\left(t\left(i\right)\right)\right)}^{2}}$$ (3)

Where:

*q’*_*j*_*(t(i))*: generalized coordinates before optimization

*q*_*j*_*(t(i))*: generalized coordinates after optimization

Also, the root mean square value of the Cartesian distance between markers before and after optimization were calculated:

$$RMS_{s,k}=\sqrt{\frac{1}{N}\cdot \sum_{i=1}^{N}{|{\bar{s}}_{k}\left(t\left(i\right)\right)-\bar{s}{'}_{k}\left(t\left(i\right)\right)|}^{2}}$$ (4)

Where:

$$\bar{s}{'}_{k}\left(t\left(i\right)\right):\;\mathrm{marker}\;\mathrm{position}\;\mathrm{before}\;\mathrm{optimization}$$

$${\bar{s}}_{k}\left(t\left(i\right)\right):\;\mathrm{marker}\;\mathrm{position}\;\mathrm{after}\;\mathrm{optimization}$$

*i*: time index

*j* and *k*: indexes for the for the generalized coordinate and the marker respectively.

The optimized net joint torques *T*_*m*_*(t(i))* were compared to the classic joint torques *T’*_*m*_*(t(i))* by a relative measure (joints indexed by *m*):

$$RMS_{Trel,m}=\frac{\sqrt{\frac{1}{N}\cdot \sum_{i=1}^{N}{\left(T_{m}\left(t\left(i\right)\right)-T{'}_{m}\left(t\left(i\right)\right)\right)}^{2}}}{\sqrt{\frac{1}{N}\cdot \sum_{i=1}^{N}T{'}_{m}{\left(t\left(i\right)\right)}^{2}}}$$ (5)

Subsequently, the grand mean and standard deviations of these RMS values were calculated over all strides. For individual trials the net joint power before and after optimization was calculated as the scalar product of joint torque and joint angular velocity, whereas power by the residual torque was calculated as the scalar product of residual torque and the trunk’s angular velocity. Power of the residual force, applied to the shoulder, was calculated as the dot product of the residual force and the velocity of its point of application and was added to the power of the residual torque.

## Supporting information

S1 File Matlab file for typical example. (7Z)
